# Spatial and temporal heterogeneity of tumor immune microenvironment between primary tumor and brain metastases in NSCLC

**DOI:** 10.1186/s12885-024-11875-w

**Published:** 2024-01-24

**Authors:** Jin-Sheng Liu, Yu-Xiang Cai, Yong-Ze He, Jian Xu, Su-Fang Tian, Zhi-Qiang Li

**Affiliations:** 1 Department of Neurosurgery, Zhongnan Hospital of Wuhan University, 430062 Wuhan, China; 2 Department of Pathology, Zhongnan Hospital of Wuhan University, 430062 Wuhan, China

**Keywords:** Non-small cell lung cancer, Brain metastases, Immune checkpoint, Tumor microenvironment, Immunotherapy

## Abstract

**Background:**

Brain metastasis is a common outcome in non-small cell lung cancer, and despite aggressive treatment, its clinical outcome is still frustrating. In recent years, immunotherapy has been developing rapidly, however, its therapeutic outcomes for primary lung cancer and brain metastases are not the same, suggesting that there may be differences in the immune microenvironment of primary lung cancer and brain metastases, however, we currently know little about these differences.

**Methods:**

Seventeen paired samples of NSCLC and their brain metastases and 45 other unpaired brain metastases samples were collected for the current study. Immunohistochemical staining was performed on all samples for the following markers: immune checkpoints CTLA-4, PD-1, PD-L1, B7-H3, B7-H4, IDO1, and EphA2; tumor-infiltrating lymphocytes (TILs) CD3, CD4, CD8, and CD20; tumor-associated microglia/macrophages (TAMs) CD68 and CD163; and tumor proliferation index Ki-67. The differences in expression of these markers were compared in 17 paired samples, and the effect of the expression level of these markers on the prognosis of patients was analyzed in lung adenocarcinoma brain metastases samples. Subsequently, multiplex immunofluorescence staining was performed in a typical lung-brain paired sample based on the aforementioned results. The multiplex immunofluorescence staining results revealed the difference in tumor immune microenvironment between primary NSCLC and brain metastases.

**Results:**

In 17 paired lesions, the infiltration of CTLA-4^+^ (*P* = 0.461), PD-1^+^ (*P* = 0.106), CD3^+^ (*P* = 0.045), CD4^+^ (*P* = 0.037), CD8^+^ (*P* = 0.008), and CD20^+^ (*P* = 0.029) TILs in brain metastases were significantly decreased compared with primary tumors. No statistically significant difference was observed in the CD68 (*P* = 0.954) and CD163 (*P* = 0.654) TAM infiltration between primary NSCLC and paired brain metastases. In all the brain metastases lesions, the expression of PD-L1 is related to the time interval of brain metastases in NSCLC. In addition, the Cox proportional hazards regression models showed high expression of B7-H4 (hazard ratio [HR] = 3.276, 95% confidence interval [CI] 1.335–8.041, *P* = 0.010) and CD68 TAM infiltration (HR = 3.775, 95% CI 1.419–10.044, *P* = 0.008) were independent prognosis factors for lung adenocarcinoma brain metastases patients.

**Conclusions:**

Both temporal and spatial heterogeneity is present between the primary tumor and brain metastases of NCSLC. Brain metastases lesions exhibit a more immunosuppressive tumor immune microenvironment. B7-H4 and CD68^+^ TAMs may have potential therapeutic value for lung adenocarcinoma brain metastases patients.

**Supplementary Information:**

The online version contains supplementary material available at 10.1186/s12885-024-11875-w.

## Background

Brain metastases have become the leading cause of death in patients with non-small cell lung cancer (NSCLC), occurring approximately in 30–50% of patients with NSCLC [1]. Furthermore, with the ageing of the population, rise of people’s living standards, and improvements in imaging technology, an increasing number of individuals are diagnosed with brain metastases. The prognosis of patients with NSCLC brain metastases is dismal, with a median survival of 7 months [2]. Therapeutic methods for patients with NSCLC brain metastases are limited. Standard treatments include surgical resection, whole-brain radiotherapy, and stereotactic radiosurgery. However, despite active treatment, the prognosis remains poor [3].

Cancer immunotherapy has advanced significantly in recent years. A reciprocal effect is observed between the human immune system and cancer cells, and regulating immune responses at tumor sites is a crucial mechanism for tumor immune evasion [4, 5]. The cytotoxic T-lymphocyte antigen 4 (CTLA-4) and programmed cell death protein 1 (PD-1)/programmed death ligand 1 (PD-L1) pathways, also known as an immune checkpoint, are recognized as an essential immunosuppressive mechanism in various tumors [6, 7]. Moreover, results from multiple clinical trials have confirmed the significant therapeutic effects of anti-CTLA-4 and anti-PD-1/PD-L1 inhibitors on NSCLC [8–12]. The discovery and clinical application of immune checkpoint inhibitors is a new direction in the treatment of NSCLC.

However, using immune checkpoint inhibitors have several issues. First, the use of immune checkpoint inhibitors is only effective for particular patients. Not all patients express CTLA-4 and PD-1/PD-L1 in their lesions, rendering immunotherapy less effective [13, 14]. In addition, for patients with NSCLC brain metastases, owing to the existence of the blood-brain barrier (BBB) and the distinctive immune environment of the brain, heterogeneity may exist in the tumor immune microenvironment between the primary tumor and metastases [15]. These reasons may lead to different therapeutic effects of immune checkpoint inhibitors on primary tumors and metastases.

To address these issues, we turned our attention to several other immune checkpoints than CTLA-4 and PD-1/PD-L1 that may be useful for treatment, and we compared the differences in the tumor immune microenvironment between primary and metastatic lesions in patients with NSCLC brain metastases to find therapeutic methods that benefit the patients.

B7-H3 and B7-H4 in the B7 family, indoleamine 2,3-dioxygenase 1 (IDO1), and EphA2 play important roles in immune suppression and immune escape, preventing the proliferation and activation of T cells. EphA2 also promotes the proliferation and migration of malignant tumor cells [16–23]. Therefore, B7-H3, B7-H4, IDO1, and EphA2 may be potential targets for immunotherapy. However, only a few studies are available on these molecules in patients with brain metastases from NSCLC. Knowledge about their expression levels in brain metastases lesions and whether their interaction with tumor immune microenvironment is related to the prognosis of NSCLC brain metastases patients is inadequate.

Herein, immunohistochemical investigation was performed on resected primary NSCLC and its brain metastases to analyze the differences in the tumor immune microenvironment. We also studied whether these differences were affected by the time interval of metastasis. In addition, survival analyzes were performed in lung adenocarcinoma brain metastases patients.

## Materials and methods

### Patients and tissue microarrays

Samples were obtained from the Department of Pathology, Zhongnan Hospital of Wuhan University, involving the patients with NSCLC brain metastases who underwent tumor resection at the Department of Neurosurgery and Department of Thoracic Surgery, Zhongnan Hospital of Wuhan University, between January 2016 and March 2021. The inclusion criteria for cases are as follows: (1) Aged between 18 and 80 years old; (2) Brain lesions confirmed by histopathology to be brain metastases from NSCLC (including lung adenocarcinoma, lung squamous cell carcinoma and lung large cell carcinoma); (3) Available formalin-fixed and paraffin-embedded (FFPE) tumor tissue. Patients who had received other treatments for brain metastasis before surgery and those with a history of other malignancies were excluded. The clinicopathological information of patients was collected from electronic records and pathology reports. Finally, we collected FFPE primary NSCLC tumor samples (*n* = 17), their paired brain metastases tissue (*n* = 17), and unpaired brain metastases tissue (*n* = 45) from 62 NSCLC patients with brain metastases. Among them, lung cancer samples and brain metastasis samples taken from the same patient are defined as paired sample, seventeen paired samples are from 17 different patients, and 45 unpaired brain metastasis tissues are from another 45 patients with NSCLC brain metastases. Waived consent was obtained from all patients. The study was approved by the Ethics Committee, Zhongnan Hospital of Wuhan University (ethics No. 2,019,048).

Tissue microarrays were performed following standard methods [24]. In brief, tumor core regions were marked on slides stained with hematoxylin and eosin (H&E) and prepared for tissue microarrays construction. A 1-mm tumor core region was selected with a needle from surgically resected FFPE tumor samples as representative tumor regions, and the removed tumor core regions were arrayed in blank recipient paraffin blocks.

### Immunohistochemistry staining (IHC)

Four-micrometer-thick FFPE serial sections were obtained from the tissue microarrays blocks. IHC staining of B7-H3, B7-H4, IDO1, and EphA2 was conducted as follows: The sections were dewaxed in xylene and rehydrated in alcohol. Thereafter, these were put in a boiling pressure cooker with EDTA antigen repair buffer for 150 s for antigen retrieval. Next, sections were incubated with 3.0% hydrogen peroxide solution for 15 min to block endogenous peroxidase activity. Then, sections were washed and blocked for 30 min with 5% goat serum. The primary antibody was added dropwise to the sections, and the sections were placed at 4 °C and incubated overnight. The sections were then incubated with the secondary antibody at room 37 °C for 30 min. Diaminobenzidine (DAB) was used for the chromogenic reaction. Finally, sections were counterstained with hematoxylin, and blue reaction was performed with 0.02% ammonia water, followed by rinsing with water, dehydrating with graded alcohol, and fixing with neutral gum sealing slides. In addition, immunohistochemical staining of CTLA-4, PD-1, PD-L1, CD3, CD4, CD8, CD20, CD68, CD163 and Ki-67 was performed on a Leica Bond Max automated stainer (Leica) according to the manufacturer’s protocol. Human tonsil tissue was included as a positive control. Use PBS rather than primary antibody as negative control. All samples were stained in one run.

The primary antibodies used for immunohistochemistry are as follows: B7-H3 (1:200 dilution, D9M2L, Cell Signaling Technology), B7-H4 (1:150 dilution, D1M8I, Cell Signaling Technology), IDO1 (1:200 dilution, SP260, Abcam), EphA2 (1:100 dilution, SC-398,832, Santa Cruz Biotechnology), CTLA-4 (UMAB249, ready-to-use, Zhongshan Golden Bridge Biotechnology), PD-1 (UMAB199, ready-to-use, Zhongshan Golden Bridge Biotechnology), PD-L1 (1:200 dilution, E1L3N, Cell Signaling Technology), Ki-67 (MIB1, ready-to-use, Zhongshan Golden Bridge Biotechnology), CD3 (F7.2.38, prediluted, Dako), CD4 (4B12, prediluted, Dako), CD8 (C8/144B, prediluted, Dako), CD20 (L26, prediluted, Leica), CD68 (KP1, prediluted, Dako) and CD163 (MRQ-26, prediluted, Dako). Anti-rabbit and anti-mouse secondary antibodies were acquired from Zhongshan Golden Bridge Biotechnology (Beijing, China).

### Multiplex immunofluorescence staining

As described in a previous report [25], multiplex immunofluorescence staining was performed using the Opal 7-Color IHC Kit (PerkinElmer, Waltham, MA, USA) in the FFPE tissue sections of a typically paired NSCLC and its brain metastases. The stained slides were scanned by a Vectra 3.0 multispectral imaging system (PerkinElmer, Waltham, MA, USA). The immunofluorescence markers consisted of B7-H4 (1:150 dilution, clone D1M8I, Cell Signaling Technology), CD3 (F7.2.38, prediluted, Dako), CD8 (C8/144B, prediluted, Dako), CD20 (L26, ready-to-use, Leica), CD68 (KP1, prediluted, Dako), and CK (AE1/AE3, prediluted, Dako). DAPI was used for nuclei highlighting. After dewaxing and rehydration, antigen retrievals were performed using a Meidi microwave (Meidi, China). For each primary antibody, tyramide signal amplification linked to specific fluorochrome from the multiplex immunofluorescence staining Kit was used for incubation and visualization. We performed the complete multiplex immunofluorescence staining procedure following the manufacturer’s instructions. In addition, human tonsil FFPE tissues were analyzed with and without primary antibodies following the same multiplex immunofluorescence staining procedure to establish positive and negative (autofluorescence) controls.

### IHC expression scoring

The immunohistochemical staining results were assessed using semiquantitative methods by two trained pathologists blinded to the patients’ clinical data. As no standard scoring system is available for the immunohistochemical staining results of these antibodies, we assessed each sample in terms of the degree of staining and proportion of positive tumor cells. Based on the degree of staining, they were classified into four semiquantitative groups: no positive staining (score 0), weak (score 1), moderate (score 2), and strong (score 3). The proportion of tumor cells stained was also classified into four categories: ≤25% (score 1), 26-50% (score 2), 51–75% (score 3), and > 75% (score 4). Next, the two scores were multiplied to obtain the final immunohistochemical staining score. The median of the final score was considered as the cutoff value to decide high and low expression classification. PD-L1 expression was assessed by tumor proportion score (TPS), and PD-L1 TPS ≥ 1% was considered positive, consistent with other studies [26, 27]. The number of TILs and TAMs was counted and averaged over three high-powered fields [28], and the median of the counts was considered as the cutoff value to decide high and low expression classification (since CTLA-4 and PD-1 are also expressed in lymphocytes, we also used this method for counting them). We selected 50% as the cutoff value for the Ki-67 labeling index (Supplementary Fig. 1 shows the typical immunohistochemical images of Ki-67). After careful review and discussion of all the slides by two pathologists, each slide was scored consistently. Specific results regarding scoring can be found in Supplementary Table 1.

### Statistical analysis

As described in previous studies, the expression of categorical variables between matched lesions was evaluated using agreement statistics (κ coefficient) [28, 29]. Regarding continuous variables, the normality was evaluated using the Shapiro–Wilk normality test. Paired Student’s t-test or Wilcoxon matched-pairs signed rank test was used to assess the significance of differences between paired lesions. Mann-Whitney U Test, Student’s T-Test, Fisher Test, or Chi-Square test were used to analyze the correlation between biomarkers and clinicopathological features. The Kaplan–Meier method was used for survival analysis. Cox proportional hazards model was used to determine independent prognostic variables. Overall survival (OS) was measured from the date of brain metastases lesion excision until death or the last follow-up. *P*-value < 0.05 was considered statistically significant. All statistical analyses were performed using GraphPad Prism version 8.0.1, SPSS version 26 and R version 3.6.3.

## Results

### Clinicopathological features of patients

Sixty-two cases of NSCLC with brain metastases were collected for the current study. The clinical and demographic information is shown in Table 1. Among them, primary lung cancer FFPE and their paired brain metastases FFPE were available in 17 patients (15 adenocarcinomas and two squamous carcinomas), and the other 45 patients had only brain metastases FFPE available (42 adenocarcinomas, one squamous carcinoma, one large cell carcinoma, and one large cell neuroendocrine carcinoma). Patients’ median age was 60 years (IQR 53–65 years, range 39–72 years). Forty-three patients were diagnosed with synchronous brain metastases (diagnostic time interval ≤ 1 month), and other 19 patients had metachronous brain metastases (diagnostic time interval > 1 month), and the median interval between the diagnosis of 19 patients with metachronous brain metastases was 34 months (IQR 21–54 months, range 2–180 months). Among the 17 paired patients, there were 10 synchronous metastasis patients and 7 metachronous metastasis patients.

**Table 1:** Clinicopathological features of the patients

Characteristic	n (%) or median (IQR)		
	Total	Paired	Unpaired
n	62	17	45
Gender			
female	18 (29%)	4 (23.5%)	14 (31.1%)
male	44 (71%)	13 (76.5%)	31 (68.9%)
Age at diagnosis			
<60	30 (48.4%)	9 (52.9%)	21 (46.7%)
≥ 60	32 (51.6%)	8 (47.1%)	24 (53.3%)
Smoking history			
yes	35 (56.5%)	12 (70.6%)	23 (51.1%)
no	27 (43.5%)	5 (29.4%)	22 (48.9%)
Stage at diagnosis			
I	4 (6.5%)	1 (5.9%)	3 (6.7%)
II	2 (3.2%)	1 (5.9%)	1 (2.2%)
III	4 (6.5%)	3 (17.6%)	1 (2.2%)
IV	44 (71.0%)	10 (58.8%)	34 (75.6%)
Unknown	8 (12.9%)	2 (11.8%)	6 (13.3%)
Histology			
Adenocarcinoma	57 (91.9%)	15 (88.2%)	42 (93.3%)
Squamous carcinoma	3 (4.8%)	2 (11.8%)	1 (2.2%)
Large cell carcinoma	2 (3.2%)	0 (0%)	2 (4.4%)
Diagnostic time interval			
≤ 1 month	43 (69.4%)	10 (58.8%)	33 (73.3%)
> 1 month	19 (30.6%)	7 (41.2%)	12 (26.7%)
Brain tumor size (mm), median (IQR)	29.5 (22.125, 41.75)	28.0 (16.0, 33.0)	30.0 (23.0, 42.0)
Brain metastases number			
Single brain metastases	39 (62.9%)	11 (64.7%)	28 (62.2%)
Multiple brain metastases	23 (37.1%)	6 (35.3%)	17 (37.8%)
Extracranial metastases			
Yes	13 (21%)	4 (23.5%)	9 (20.0%)
No	49 (79%)	13 (76.5%)	36 (80.0%)
EGFR Mutation			
Yes	13 (20.7%)	4(23.5%)	9 (20.0%)
No	14 (22.6%)	2(11.8%)	12 (26.7%)
Not tested	35 (56.5%)	11(64.7%)	24 (53.3%)
ALK rearranged			
Yes	1(1.6%)	0 (0%)	1(2.2%)
No	16(25.8%)	4 (23.5%)	12 (26.7%)
Not tested	45 (72.6%)	13 (76.5%)	32(71.1%)
Treatment modality			
SR	5 (8.1%)	2 (11.8%)	3 (6.7%)
SR + Rad/Chemo	18(29.0%)	7 (41.2%)	11 (24.4%)
SR + Rad/Chemo + TKIs	16(25.8%)	5 (29.4%)	11(24.4%)
Unknown	23(37.1%)	3 (17.6%)	20(44.4%)

SR: surgical resection, Rad: radiation therapy, Chemo: chemotherapy, TKIs: tyrosine kinase inhibitors

### Comparison of tumor microenvironment between primary NSCLC and paired brain metastases

**Fig. 1:**
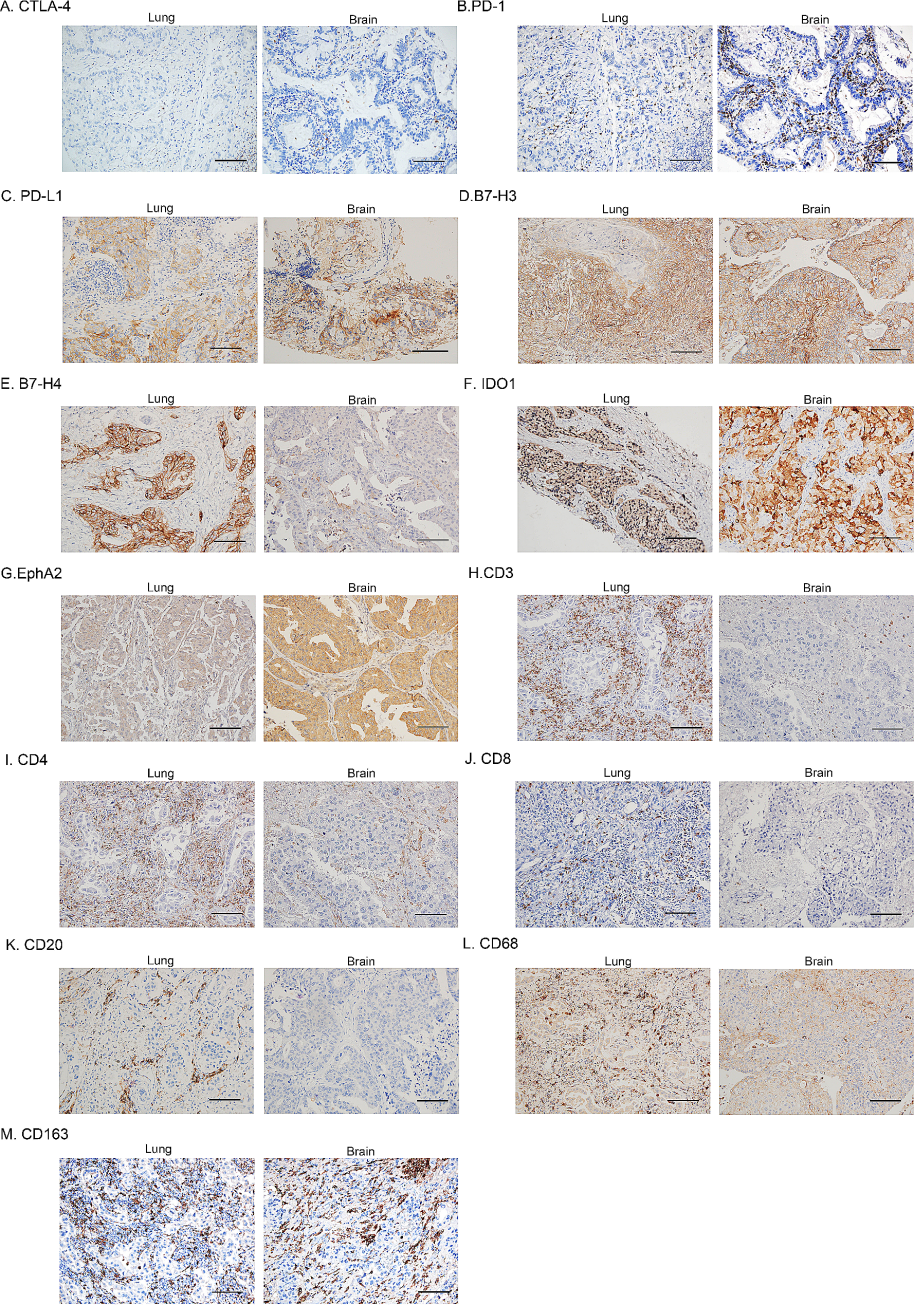
Representative immunohistochemical staining image of NSCLC and its paired brain metastases (not from the same patients). A–M list the typical immunohistochemical images of CTLA-4, PD-1, PD-L1, B7-H3, B7-H4, IDO1, EphA2, CD3, CD4, CD8, CD20, CD68, and CD163, respectively. Scale bars, 100 μm, Original magnification 200×

We compared the tumor immune microenvironment of 17 primary NSCLCs and their paired brain metastases. Figure 1 shows typical immunohistochemical images of paired lesions. The expression of PD-L1, B7-H3, B7-H4, IDO1, and EphA2 were inconsistent in the paired samples of 11.8%, 29.4%, 23.5%, 29.4%, and 52.9%, respectively. According to the agreement statistics [28, 29], PD-L1 (κ = 0.61, 95% CI 0.13–1.00, *P* = 0.007) and B7-H4 (κ = 0.53, 95% CI 0.15–0.92, *P* = 0.024) had moderate consistency in primary NSCLC and its brain metastases, and the expressions of B7-H3 (κ = 0.43, 95% CI 0.03–0.83, *P* = 0.059), IDO1 (κ = 0.38, 95% CI − 0.07–0.83, *P* = 0.115), and EphA2 (κ = − 0.04, 95% CI − 0.49–0.41, *P* = 0.858) in primary NSCLC and its brain metastases were not consistent (Details are shown in Table 2).

**Table 2:** Expression and agreement statistics (κ coefficient) of PD-L1, B7-H3, B7-H4, IDO1, and EphA2 in primary NSCLC and its brain metastases

		Brain:		Kappa data	*P*-value
		PD-L1 Positive	PD-L1 Negative		
Lung:	PD-L1 Positive	2	2	0.61	0.007
	PD-L1 Negative	0	13		
		B7-H3 high expression	B7-H3 low expression		
	B7-H3 high expression	6	1	0.43	0.059
	B7-H3 low expression	4	6		
		B7-H4 high expression	B7-H4 low expression		
	B7-H4 high expression	7	1	0.53	0.024
	B7-H4 low expression	3	6		
		IDO1 high expression	IDO1 low expression		
	IDO1 high expression	4	2	0.38	0.115
	IDO1 low expression	3	8		
		EphA2 high expression	EphA2 low expression		
	EphA2 high expression	3	3	-0.04	0.858
	EphA2 low expression	6	5		

**Fig. 2:**
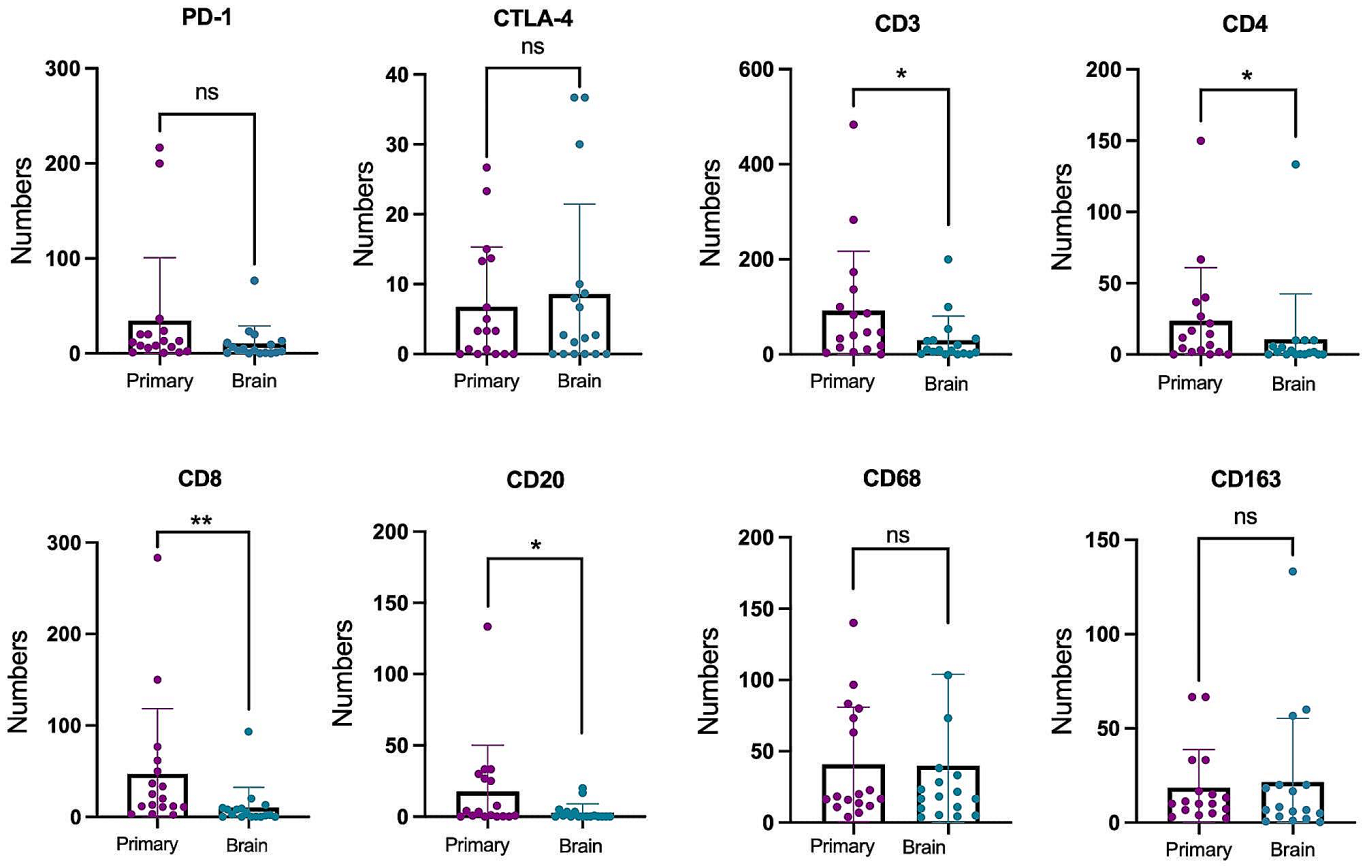
Comparison of TILs and TAMs between primary NSCLC and brain metastases lesions. Significant differences were observed in CD3, CD4, CD8, and CD20 expression levels (Numbers/HPF) between the primary NSCLC and paired brain metastases (*P* = 0.045, *P* = 0.037, *P* = 0.008, and *P* = 0.029, respectively). No significant differences were observed in the PD-1, CTLA-4, CD68 and CD163 expression levels (Numbers/HPF) between the primary NSCLC and paired brain metastases (*P* = 0.106, *P* = 0.461, *P* = 0.954 and *P* = 0.654, respectively). Differences of TILs and TAMs between primary lung cancer and brain metastasis were analyzed by the Paired Student’s t-test or Wilcoxon matched-pairs signed rank test on normality or non-normality variables, respectively. *, *P* < 0.05; **, *P* < 0.01; ns, not significant. ***HPF*** high power field

We also compared TILs and TAMs in paired lesions. As shown in Fig. 2, in primary NSCLC and paired brain metastases, the median of PD-1^+^ TILs were 11.7 (IQR 5.7–20) and 3.3 (IQR 0.7–11.3) (*P* = 0.106), the median of CD3^+^ TILs were 46.7 (IQR 12.5–118.4) and 9.0 (IQR 1.5–31.7) (*P* = 0.045), median of CD4^+^ TILs were 11.7 (IQR 1.7–31.7) and 1.7 (IQR 0.0–7.8) (*P* = 0.037), median of CD8^+^ TILs were 20.0 (IQR 11.0–55.9) and 2.7 (IQR 0.0–9.7) (*P* = 0.008), and median of CD20^+^ TILs were 3.3 (IQR 0.2–28.4) and 0.0 (IQR 0.0–3.3) (*P* = 0.029). The CTLA-4^+^ TILs in the primary NSCLC and brain metastases were 6.765 ± 8.555 and 8.600 ± 12.854 per high-power field (*P* = 0.461, a paired Student’s t-test). The CD68^+^ TAMs in the primary NSCLC and brain metastases were 40.824 ± 40.233 and 39.951 ± 64.044 per high-power field (*P* = 0.954, a paired Student’s t-test). The CD163^+^ TAMs in the primary NSCLC and brain metastases were 18.506 ± 20.242 and 21.471 ± 33.909 per high-power field (*P* = 0.654, a paired Student’s t-test). Except for special notes, all the aforementioned statistical methods are Wilcoxon matched-pairs signed rank tests. The expression of PD-1^+^ TILs, CTLA-4^+^ TILs, CD68^+^ TAMs and CD163^+^ TAMs in brain metastases tissue did not substantially differ from those in primary NSCLC (*P* = 0.106, *P* = 0.461, *P* = 0.954 and *P* = 0.654, respectively). In addition, interestingly, the expression of CD3^+^, CD4^+^, CD8^+^, and CD20 + TILs in brain metastases were significantly decreased compared with primary tumors (*P* = 0.045, *P* = 0.037, *P* = 0.008, and *P* = 0.029, respectively).

**Fig. 3:**
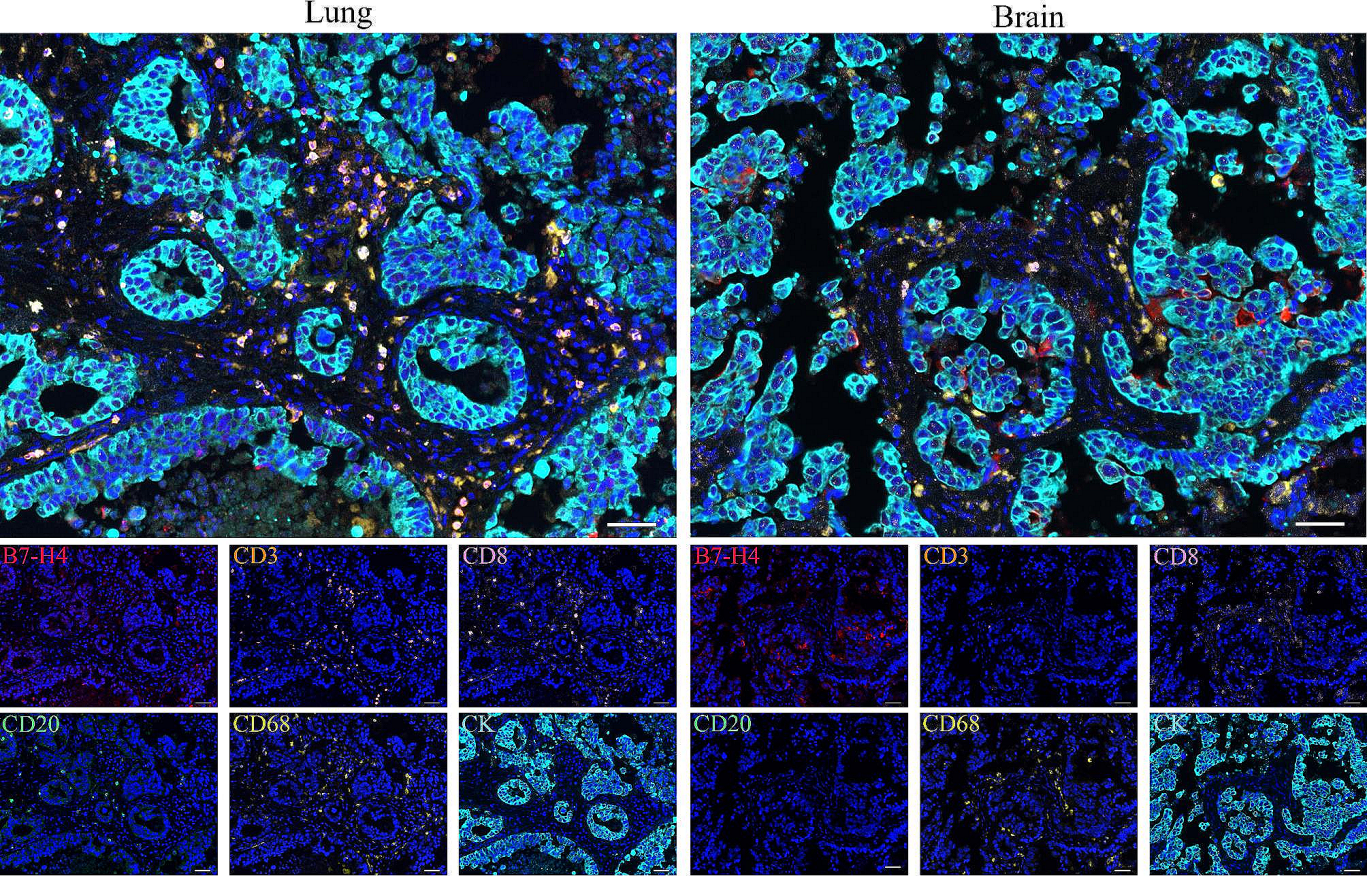
Representative images of multiplex immunofluorescence in primary lung cancer and brain metastasis tissues. Scale bars, 50 μm

Based on the results of subsequent prognostic analysis, we selected B7-H4, CD3, CD8, CD20, and CD68 for multiplex immunofluorescence staining to show more straightforwardly the differences in the tumor immune microenvironment of the primary tumor and brain metastases. The tumor epithelial cells and nuclei were labeled with CK and DAPI, respectively (Fig. 3). We observed prominent colocalization of B7-H4 with CK (tumor cell marker), indicating that B7-H4 is mainly expressed in tumor cells. CD3, CD8, and CD20 were expressed in the tumor stroma, and significantly more lymphocyte infiltration was observed in the primary tumor compared with brain metastases. CD68^+^ TAMs were expressed in the tumor stroma of both primary tumors and brain metastases, and no significant difference was observed between the expression levels.

### Difference in tumor microenvironment between synchronous and metachronous NSCLC brain metastases

**Fig. 4:**
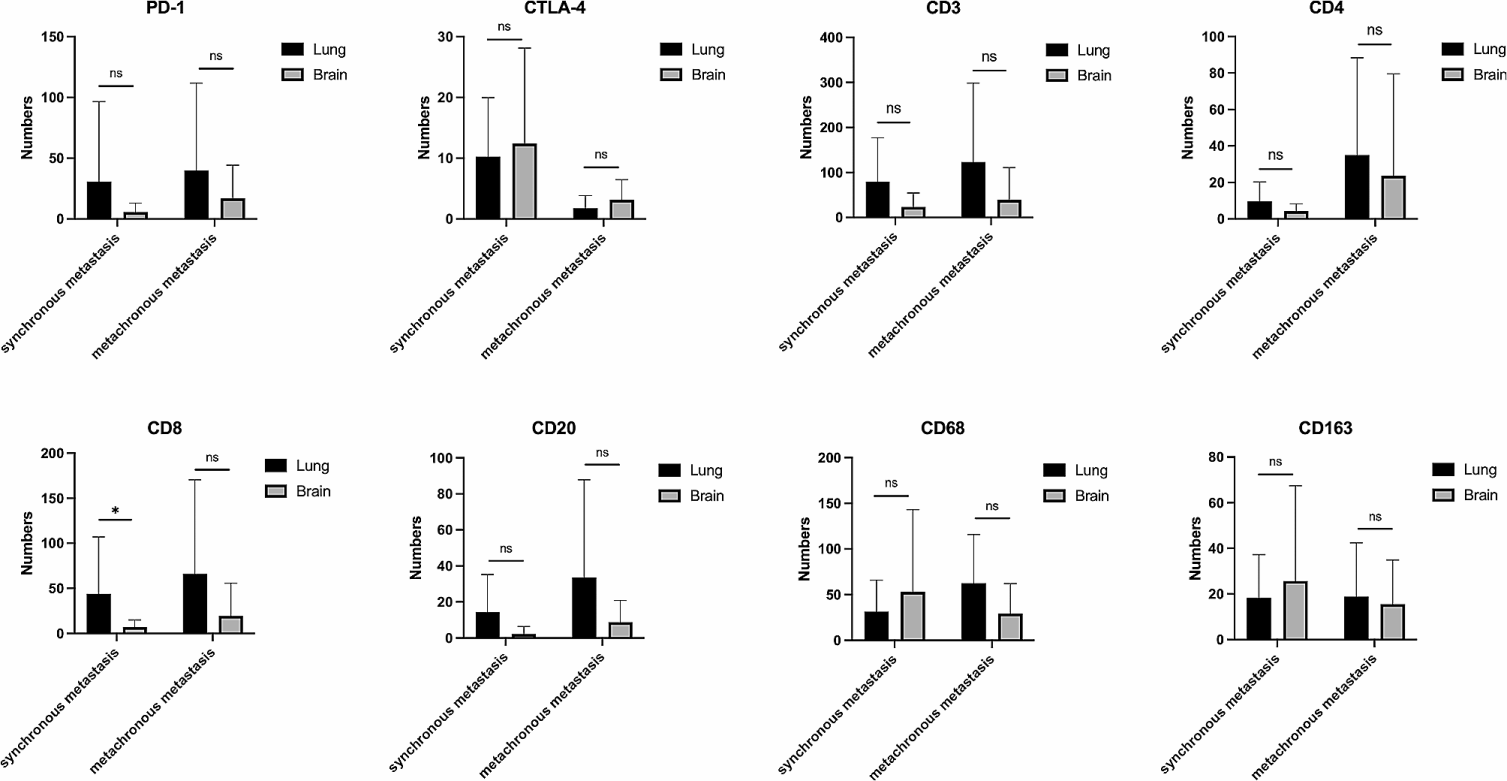
Infiltration differences of TILs in synchronous and metachronous brain metastases patients. The infiltration of CD8^+^ lymphocytes were remarkably decreased in synchronously metastatic brain lesions (*P* = 0.034). No significant difference was observed between the paired lesions of synchronous and metachronous metastases among the other markers. *, *P* < 0.05; ns, not significant. The significance in difference was analyzed by Mann-Whitney U Test

**Fig. 5:**
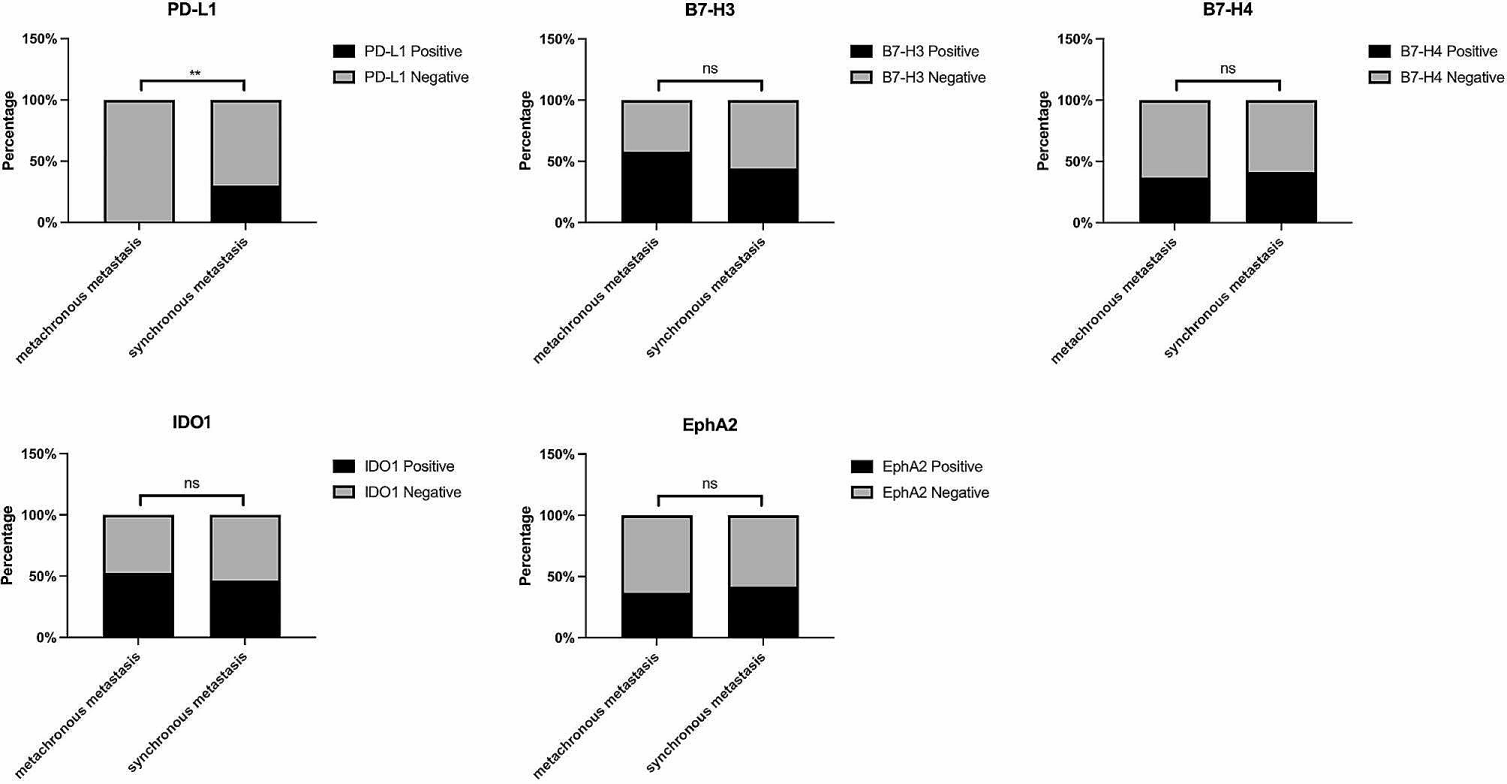
The expression difference of PD-L1 (*P* = 0.006), B7-H3 (*P* = 0.471), B7-H4 (*P* = 0.956), IDO1 (*P* = 0.866) and EphA2 (*P* = 0.928) in synchronous and metachronous brain metastases. **, *P* < 0.01; ns, not significant. The significance in difference was analyzed by Chi-Square test

All the samples were divided into two groups: synchronous metastasis and metachronous metastasis. Among 17 paired samples, the infiltration of CD8^+^ lymphocytes were remarkably decreased in synchronously metastatic brain lesions (*P* = 0.034, Fig. 4). No significant difference was observed between the paired lesions of synchronous and metachronous metastases among the other markers (Fig. 4, data details can be found in Supplementary Table 2). In addition, in 62 cases of brain metastases lesions, positive PD-L1 expression was significantly related to synchronous diagnosis of primary tumor and brain metastases (*P* = 0.006). None of the metachronous brain metastases were PD-L1 positive, while the PD-L1 positive rate was significantly increased in synchronous brain metastases (Fig. 5). No other significant difference was observed among the brain metastases lesions of synchronous and metachronous metastases.

### Relationship between immune checkpoint expression and clinicopathological features in brain metastases

The relationship between immune checkpoints and clinicopathological features of patients was explored in their brain metastases lesions (Supplementary Table 1). The relationship between PD-L1 expression and the time interval between the diagnosis of primary and metastatic lesions has been described in the previous section. In addition, we found that B7-H3 expression was related to brain metastases size. Patients with high B7-H3 expression tended to have a smaller brain tumor, with a median size of 24 mm (IQR 19.25–34 mm) compared with 33.5 mm (IQR 25.25–45.75 mm) in the low expression group (*P* = 0.012). PD-1 expression was related to extracranial metastases (*P* = 0.029). No significant correlation was observed between CTLA-4, B7-H4, IDO1, EphA2, and clinicopathological features.

### Effect of tumor microenvironment on metastatic brain tumor prognosis

**Fig. 6:**
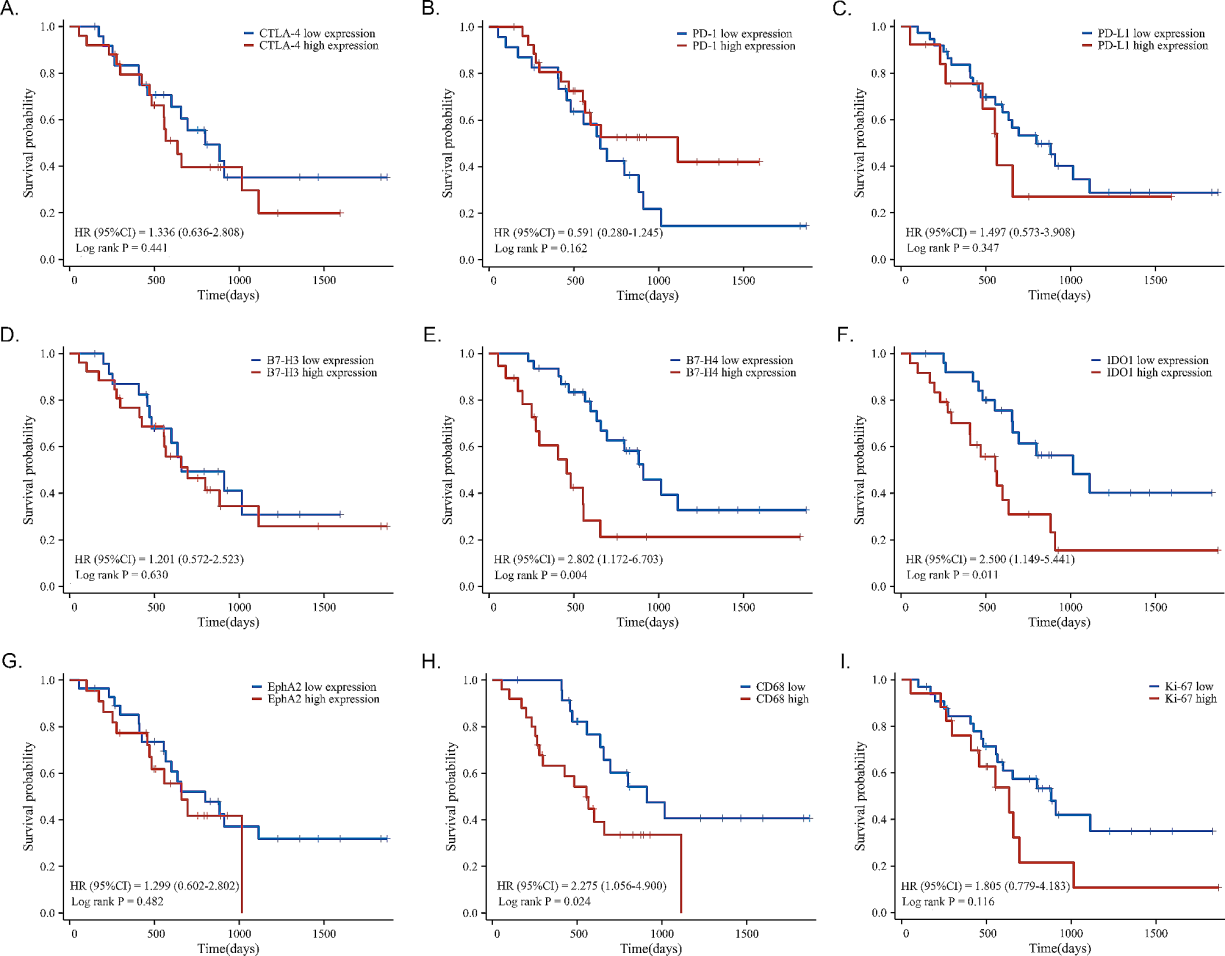
Kaplan–Meier survival curves of CTLA-4, PD-1, PD-L1, B7-H3, B7-H4, IDO1, EphA2, CD68, and Ki-67 in lung adenocarcinoma brain metastases patients. (**A-D**) CTLA-4 (*P* = 0.441), PD-1 (*P* = 0.162), PD-L1 (*P* = 0.347), and B7-H3 (*P* = 0.630) expression was not correlated with patient survival. (**E** and **F**) High expression of B7-H4 and IDO1 in tumor cells was associated with worse survival (*P* = 0.004 and *P* = 0.011, respectively). (**G**) EphA2 expression in tumor cells was not associated with patient survival (*P* = 0.482). (**H**) High CD68^+^ cells in the stroma were associated with worse survival (*P* = 0.024). (**I**) Ki-67 index was not associated with patient survival (*P* = 0.116). The significance in survival differences was analyzed by log-rank test

We explored the relationship between the expression of immune checkpoints, lymphocyte infiltration, and patient prognosis in brain metastatic lesions (Supplementary Fig. 2 shows typical immunohistochemical images of all markers in brain metastases). High levels of B7-H3, B7-H4, IDO1, and EphA2 expression in lung cancer are correlated to a poor prognosis [30–33]. However, the impact of their expression on OS in patients with NSCLC brain metastases has rarely been mentioned. Among the total 62 patients in this study, compared with lung adenocarcinoma, the number of patients with lung squamous cell carcinoma and large cell carcinoma is smaller, and their immune microenvironment and prognosis are highly heterogeneous [34]. In order to obtain more accurate results, we excluded these patients (3 squamous cell carcinoma patients, 2 large cell carcinoma patients) in the survival analysis. Besides, seven patients were lost to follow-up, so we only included 50 patients with follow-up information in the final survival analysis. The median observation period is 793 (range 147–1870) days. Results revealed that patients with high B7-H4 (hazard ratio [HR] = 2.802, 95% confidence interval [CI] 1.172–6.703, *P* = 0.004, Fig. 6E) and IDO1 (HR = 2.500, 95% CI 1.149–5.441, *P* = 0.011, Fig. 6F) expression in brain metastases had significantly decreased OS. Nevertheless, the expression of PD-L1 (HR = 1.497, 95% CI 0.573–3.908, *P* = 0.347, Fig. 6C), B7-H3 (HR = 1.201, 95% CI 0.572–2.523, *P* = 0.630, Fig. 6D) and EphA2 (HR = 1.299, 95% CI 0.602–2.802, *P* = 0.482, Fig. 6G) in NSCLC brain metastases patients did not have a significant relationship with the OS.

Furthermore, the effect of TILs and TAMs in the immune microenvironment of brain metastases and Ki-67 labeling index on the OS of patients with lung adenocarcinoma brain metastases were studied. Patients with higher CD68^+^ microglia/macrophage infiltration demonstrated a worse OS (HR = 2.275, 95% CI 1.056–4.900, *P* = 0.024, Fig. 6H). The CTLA-4^+^ (HR = 1.336, 95% CI 0.636–2.808, *P* = 0.441, Fig. 6A) and PD-1^+^ (HR = 1.591, 95% CI 0.280–1.245, *P* = 0.162, Fig. 6B) lymphocyte infiltration in NSCLC brain metastases patients did not have a significant relationship with the OS. CD3^+^ (HR = 0.586, 95% CI 0.275–1.246, *P* = 0.152, Supplementary Fig. 3A), CD4^+^ (HR = 0.687, 95% CI 0.327–1.442, *P* = 0.325, Supplementary Fig. 3B), CD8^+^ (HR = 1.095, 95% CI 0.521–2.298, *P* = 0.810, Supplementary Fig. 3C), CD20^+^ (HR = 0.762, 95% CI 0.363–1.598, *P* = 0.472, Supplementary Fig. 3D) lymphocyte infiltration and CD163^+^ macrophage infiltration (HR = 1.535, 95% CI 0.730–3.226, *P* = 0.257, Supplementary Fig. 3E), CD163^+^/CD68^+^ ratio (HR = 1.661, 95% CI 0.787–3.502, *P* = 0.202, Supplementary Fig. 3F) had no prognostic significance in patients with NSCLC brain metastases. Moreover, the Ki-67 levels had no related to outcome (HR = 1.805, 95% CI 0.779–4.183, *P* = 0.116, Fig. 6I).

**Fig. 7:**
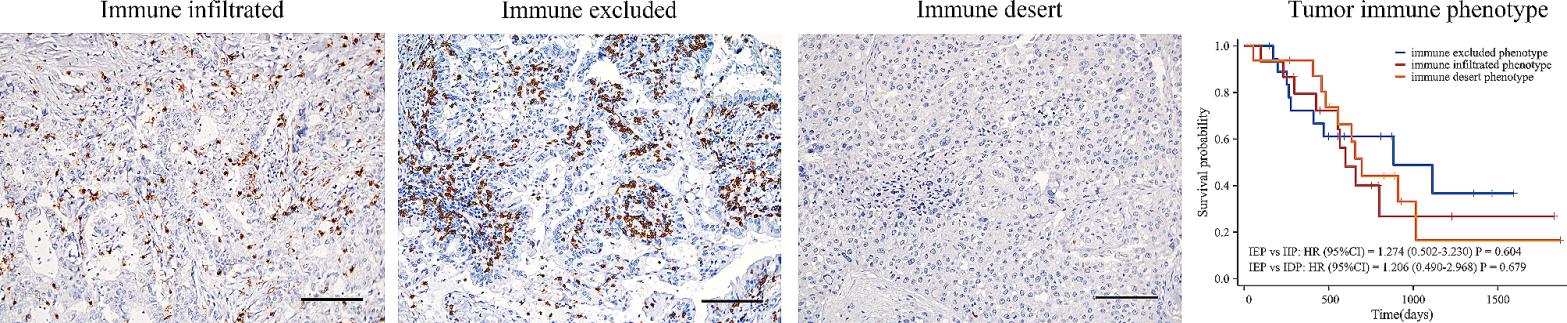
Representative pictures of different immune infiltration types and corresponding Kaplan–Meier survival curves. The significance in survival differences was analyzed by log-rank test. Scale bars, 100 μm, Original magnification 200×

Recently, some scholars have proposed that immune cells in tumors have different infiltration patterns, which may affect the prognosis of patients [35, 36]. We followed the classification rules described by them to classify patients into immune infiltration phenotype, immune excluded phenotype and immune desert phenotype, and conducted survival analysis. However, no association between immune infiltration type and patient prognosis was found in our study (immune excluded phenotype vs. immune infiltration phenotype: HR = 1.274, 95%CI = 0.502–3.230, *P* = 0.604; immune excluded phenotype vs. immune desert phenotype: HR = 1.206, 95%CI = 0.490–2.968, *P* = 0.679, Fig. 7).

Finally, the Cox proportional hazards model was used for univariate and multivariate analyses. *P* values < 0.100 in univariate analysis were considered in multivariate analysis. Since there are many missing values for the patient’s subsequent treatment modality, in order to ensure the accuracy of the results, we did not include it as a variable in the Cox proportional hazards model, but only drew the survival curve (Supplementary Fig. 4). In addition, as we know that patients with higher tumor stages often have a poorer prognosis, tumor stage was also included as a covariate in subsequent multivariate analyses (Supplementary Table 4). Hereby, age, stage at diagnosis, B7-H4, IDO1, and CD68^+^ macrophage infiltration, were included in the multivariate analysis. The findings revealed that higher expression of B7-H4 (HR = 3.276, 95% CI 1.335–8.041, *P* = 0.010) and higher CD68^+^ macrophage infiltration (HR = 3.775, 95% CI 1.419–10.044, *P* = 0.008) were independent prognostic factors for patients with NSCLC brain metastases (Table 3). However, higher expression of IDO1 (HR = 1.719, 95% CI 0.683–4.326, *P* = 0.250) was excluded as an independent prognostic factor for these patients, which may be owing to the interaction between IDO1 and other tested factors.

**Table 3:** Multivariate analysis of prognostic factor for lung adenocarcinoma brain metastases patients

Characteristics	Total(N)*	Multivariate analysis	
		HR (95% CI)	*P* value
Age at diagnosis (years)			
<60	18	Reference	
≥ 60	25	1.148 (0.434–3.036)	0.781
stage at diagnosis			
I-III	7	Reference	
IV	36	0.427 (0.136–1.345)	0.146
B7-H4			
low	27	Reference	
high	16	3.276 (1.335–8.041)	**0.010**
IDO1			
low	23	Reference	
high	20	1.719 (0.683–4.326)	0.250
CD68			
low	22	Reference	
high	21	3.775 (1.419–10.044)	**0.008**

Indicators with *P* values in bold were independent prognostic factor for lung adenocarcinoma brain metastases patients

* Because the “stage at diagnosis” of 7 patients was unknown, only 43 patients were finally included in the multivariate analysis

## Discussion

Herein, using immunohistochemistry, we discovered that the tumor microenvironment of primary NSCLC and brain metastases has both spatial and temporal heterogeneity. Spatially, the expression of some immune checkpoints was inconsistent. Besides, the infiltration of lymphocytes in brain metastases was significantly reduced compared with primary tumors, suggesting a more immunosuppressed microenvironment. Temporally, in paired lesions, CD8^+^ lymphocytes were more significantly suppressed in synchronous metastases. In brain metastases lesions, the positive expression of PD-L1 was significantly associated with synchronous metastases. In addition, we also explored the factors related to the prognosis of patients with lung adenocarcinoma brain metastases, and the results revealed the age, expression of B7-H4 and IDO1, and infiltration of CD68^+^ TAM were related to the prognosis. The expression of B7-H4 and the infiltration of CD68^+^ TAM are independent prognostic factors for lung adenocarcinoma brain metastasis patients.

The brain has been considered immune-privileged for an extended period because of the BBB. In recent years, however, many researchers have mentioned that the brain is immunologically unique instead of privileged since the discovery of functional lymphatic vessels within the central nervous system, and the immune cell infiltration of brain tumors is rare, but it occurs [15]. This echoes our findings on the spatial heterogeneity of primary NSCLC and brain metastases. We found that the TILs density in brain metastases was significantly lower than in primary tumors, which is consistent with the findings of some previous groups [37, 38]. These results partly explain why some immune checkpoint inhibitors exhibit different therapeutic responses in the intracranial and extracranial regions in patients with NSCLC brain metastases [39]. Clinicians should fully consider this before applying immunotherapy.

Only a few studies have used the time interval between lung cancer and brain metastasis as a subgroup to analyze differences in the tumor microenvironment. We found that in brain metastasis tissue, PD-L1 positivity was more common in synchronous metastases than in metachronous metastases. This is similar to the findings of Lee et al., who performed IHC of PD-L1 on primary lung cancer tissues of 270 patients with NSCLC brain metastases, and the results showed that compared with PD-L1-negative patients, synchronous brain metastases were more frequently observed in PD-L1-positive patients [40]. This indicates that PD-L1 expression is strongly correlated with synchronous metastasis whether detected in primary lung tumors or brain metastases. This finding is interesting. Is PD-L1 related to the activation of metastasis of NSCLC to the brain? The reason is still unclear. The mechanism of lung cancer brain metastasis has been reported before, including the “hemodynamic hypotheses” and “seed-and-soil hypotheses” [41, 42]. In our clinical observations, the time interval for lung cancer to metastasize to the brain considerably varies greatly. This may be related to different initiation times of metastasis, impediments in the metastasis process, or the length of latency of tumor cells in the brain. Whether these processes are affected by PD-L1 expression remains to be confirmed by further research. We also noticed that previous literature has studied the relationship between the time interval of metastasis and the tumor microenvironment (including the expression of PD-L1) in paired NSCLC and brain metastases resected lesions. Most research results show that compared with metachronous metastasis, the tumor microenvironment of the primary tumor and brain metastasis tumor of synchronous brain metastasis is more consistent and less difference [28, 43–45]. Only one literature reported different results [46]. This finding has important clinical significance. For synchronous NSCLC brain metastases, the more common treatment is surgical resection of the brain metastases without surgical intervention of the primary tumor. Because for advanced NSCLC with distant metastasis, surgical resection of the primary tumor is not considered an effective treatment method [47], which leads to a result: we can only perform pathological analysis on brain metastasis tissue, such as the expression of CTLA-4, PD-1, PD-L1, etc., rather than lung. Determining the consistency of tumor microenvironment expression between primary tumors and brain metastases can help clinicians decided whether patients are suitable for subsequent immunotherapy.

Previous studies have shown that B7-H4 and IDO1 have predictive significance in NSCLC [30, 33]. However, we know little about their relationship with the prognosis of NSCLC brain metastasis patients. We performed survival analyzes in lung adenocarcinoma brain metastases patients. The result showed patients with high B7-H4 and IDO1 expression in brain metastases had a shorter survival time (log-rank test), and B7-H4 was an independent prognostic factor in lung adenocarcinoma brain metastases patients. We noticed that a previous study also reported the effect of B7-H4 on overall survival in NSCLC brain metastasis [48]. Although the classification criteria for high B7-H4 expression in this study were different, a consistent conclusion was reached, which indicates that B7-H4 is still associated with disease prognosis even at different cutoff values and is a meaningful predictor of prognosis in NSCLC brain metastases patients. As far as we know, this study is the first to investigate the prognostic value of IDO1 in lung adenocarcinoma brain metastases. Although the final results show that IDO1 is not an independent prognostic factor, the widespread expression of IDO1 in brain metastases and its inhibitory effect on immune cells may make it a potential therapeutic target. Phase I/II clinical trials of epacadostat, an IDO1 inhibitor, have been reported positively in many advanced solid tumors [49, 50]. Despite a recent phase III ECHO-301 trial (NCT02752074) of PD-1 inhibitor in combination with an IDO1 inhibitor in metastatic melanoma showed no significant clinical benefit in the treatment group (pembrolizumab + epacadostat) compared with the control group (pembrolizumab + placebo) [51]. Some immuno-oncologists still believe that IDO1 is not a “bad target,” and the IDO1-targeting therapy is still meaningful. Vernon K. Sondak et al. explained this phenomenon to be dose-related [52]. However, the mechanism of IDO1 immunosuppression and specific biomarkers that can respond to IDO inhibitors need to be explored in future research.

The findings of the current study revealed that CD68^+^ TAM infiltration have prognostic significance in lung adenocarcinoma brain metastases. Recently, TAMs have become a hotspot in tumor immunotherapy research. According to the traditional classification, TAMs are usually divided into M1 and M2 phenotypes. M1 phenotype is considered to have pro-inflammatory and anti-tumor effects, and M2 phenotype is considered to have angiogenic and pro-tumor effects [53]. Previous research has indicated that most immune cells within primary brain tumors are macrophages, comprising approximately 30% of the tumor mass. Compared with extracranial organs, the brain as an immunologically unique organ has two sources of TAMs: brain resident microglia and bone marrow-derived macrophages, both of which can be labeled by CD68, and the latter are risen by circulating monocytes recruited into the brain in pathological conditions such as tumors [54–56]. Up to now, there is still no unified standard for how to distinguish brain resident microglia and bone marrow-derived macrophages. In this study, the high density of CD68^+^ TAM infiltration in brain lesions was confirmed to be a poor prognostic factor in patients with lung adenocarcinoma brain metastases, whereas CD163-labeled M2 macrophage infiltration and CD163/CD68 ratio were not associated with prognosis. This may be because brain resident microglia are contained within CD68-positive TAM. Previous study has shown that brain resident microglia, and not peripheral macrophages, are the main source of brain tumor mononuclear cells [57]. Interactions between microglia and T cells can promote brain cancer heterogeneity and immunosuppression [58]. This may partially explain our result. In addition, the co-expression of M1 and M2 markers and M1 and M2 phenotype switching is also existing in brain tumors [59, 60]. The immune microenvironment in brain tumors is a dynamic process. In our study, we can only confirm the status of TAM at the moment of surgical resection, and whether there is a phenotype switching between M1 and M2 after surgery is unknown. In summary, targeting intracranial microglia or M2 macrophages may be a potential treatment modality for lung adenocarcinoma brain metastases patients and more studies are needed to confirm this.

Although we did not find a significant correlation between the prognosis of patients with brain metastatic NSCLC and the expression of PD-1, PD-L1 and B7-H3 in the current study, previous studies have confirmed that PD-1, PD-L1 and B7-H3 play an important role in primary brain tumors, including glioblastoma and primary central nervous system lymphoma, and are associated with patient prognosis [61–63]. This shows that these tumor immune checkpoints still interact with the surrounding immune microenvironment in the brain, a special immune-privileged organ [15], and thus exert corresponding functions. Although brain metastatic NSCLC is a tumor of extracranial origin, it eventually colonizes and proliferates in the brain, and also has the expression of PD-1, PD-L1 and B7-H3. The specific mechanism remains to be explored. In the future, it may be meaningful to include primary brain tumors and metastatic brain tumors in one study and analyze the expression of these markers and their relationship with the immune microenvironment.

Despite providing some valuable insights, this study has several limitations. First, the small number of paired samples is a major limitation of our study. It is a single-center study and not all patients have undergone primary lesion resection at Zhongnan Hospital of Wuhan University. In addition, some patients only received resection of brain metastases. For the primary lung cancer, they did not receive surgical intervention but chose radiotherapy and\or chemotherapy. This resulted in our inability to obtain primary tumor tissue of these patients. Second, the clinical data of some patients were incomplete, affecting subsequent analysis. Third, the tumor core tissue used to make the tissue microarrays may not accurately represent the expression of markers in the panorama of tumor tissue. However, the results from the tissue microarrays measuring the expression of immune markers in several studies were consistent with each other, which justifies this approach.

## Conclusion

In summary, knowing the spatial and temporal heterogeneity of the tumor immune microenvironment of primary NSCLC and brain metastases and exploring the role of immune checkpoints in different stages of tumor evolution is crucial to identify the most appropriate treatment options. In addition, we also identified intracranial B7-H4 and CD68^+^ TAMs as two valuable predictors of prognosis in patients with NSCLC with brain metastases. This study provides a clinical basis for immunotherapy in patients with lung adenocarcinoma brain metastases. Future studies are necessary to explore the molecular mechanism and develop drugs. Multicenter large sample clinical trials need to be performed to help us better understand NSCLC brain metastases and develop new treatment methods to improve patient prognosis.

### Electronic supplementary material

Below is the link to the electronic supplementary material.

**Supplementary Material 1: Supplementary table 1.** The immunohistochemical scores and medians of immune checkpoints, tumor-infiltrating lymphocytes, tumor-associated microglia/macrophages and tumor proliferation index Ki-67. **Supplementary table 2.** Infiltration of TILs in paired samples when classified by synchronous and metachronous metastasis. **Supplementary table 3:** Relationship between the expression of immune checkpoints and clinicopathological features in BM lesions. **Supplementary table 4:** Univariate analysis of prognostic factor for lung adenocarcinoma brain metastases patients. **Supplementary Fig. 1:** Typical immunohistochemical images of Ki-67. Original magnification 200×. Scale bars, 100 µm. **Supplementary Fig. 2:** Representative immunohistochemical staining images of all markers in brain metastases. A to M list the typical weak positive expression, moderate positive expression, strong positive expression immunohistochemical images of CTLA-4, PD-1, PD-L1, B7-H3, B7-H4, IDO1, EphA2, CD3, CD4, CD8, CD20, CD68 and CD163, respectively. Original magnification 200×. Scale bars, 100 µm. **Supplementary Fig. 3:** Kaplan-Meier survival curves of CD3, CD4, CD8, CD20, CD163 and CD163/CD68 ratio in lung adenocarcinoma brain metastases patients. (A-F) the expression of CD3, CD4, CD8 CD20, CD163 and CD163/CD68 in stroma were not associated with patient survival (P = 0.152, P = 0.325, P = 0.810, P = 0.472, P = 0.257 and P = 0.202 respectively). **Supplementary Fig. 4:** Kaplan-Meier survival curves of treatment modality in lung adenocarcinoma brain metastases patients (n = 31). SR: surgical resection, Rad: radiation therapy, Chemo: chemotherapy, TKIs: tyrosine kinase inhibitors

## Data Availability

The raw data for this study will be provided by the authors upon reasonable request. Requests for access to the dataset should be sent to lizhiqiang@whu.edu.cn.

## Abbreviations

BBB: Blood–brain barrier
CI: Confidence interval
DAB: Diaminobenzidine
FFPE: Formalin-fixed and paraffin-embedded
H&E: Hematoxylin and eosin
HR: Hazard ratio
IDO1: Indoleamine 2,3-dioxygenase 1
IHC: Immunohistochemistry staining
NSCLC: Non-small cell lung cancer
OS: Overall survival
PD-1: Programmed cell death protein 1
PD-L1: Programmed death ligand 1
TAMs: Tumor-associated microglia/macrophages
TILs: Tumor-infiltrating lymphocytes
TPS: Tumor proportion score
