# Supplementary material for: Spatial and temporal heterogeneity of tumor immune microenvironment between primary tumor and brain metastases in NSCLC
Source: BMC Cancer. 2024 Jan 24;24:123. doi: 10.1186/s12885-024-11875-w (PMC10809508; doi:10.1186/s12885-024-11875-w)

Supplementary table 1.

The immunohistochemical scores and medians of immune checkpoints, tumor-infiltrating lymphocytes, tumor-associated microglia/macrophages and tumor proliferation index Ki-67

Supplementary table 2.

Infiltration of TILs in paired samples when classified by synchronous and metachronous metastasis

Supplementary table 3:

Relationship between the expression of immune checkpoints and clinicopathological features in BM lesions.

Supplementary table 4:

Univariate analysis of prognostic factor for lung adenocarcinoma brain metastases patients.

Indicators with P values in bold were included in the multivariate analysis

* It is well known that tumor stage is related to patient prognosis, so we also included it as a covariate in the subsequent multivariate analysis, although its P value > 0.100.

Supplementary figure 1:

Typical immunohistochemical images of Ki-67. Original magnification 200×. Scale bars, 100 µm.


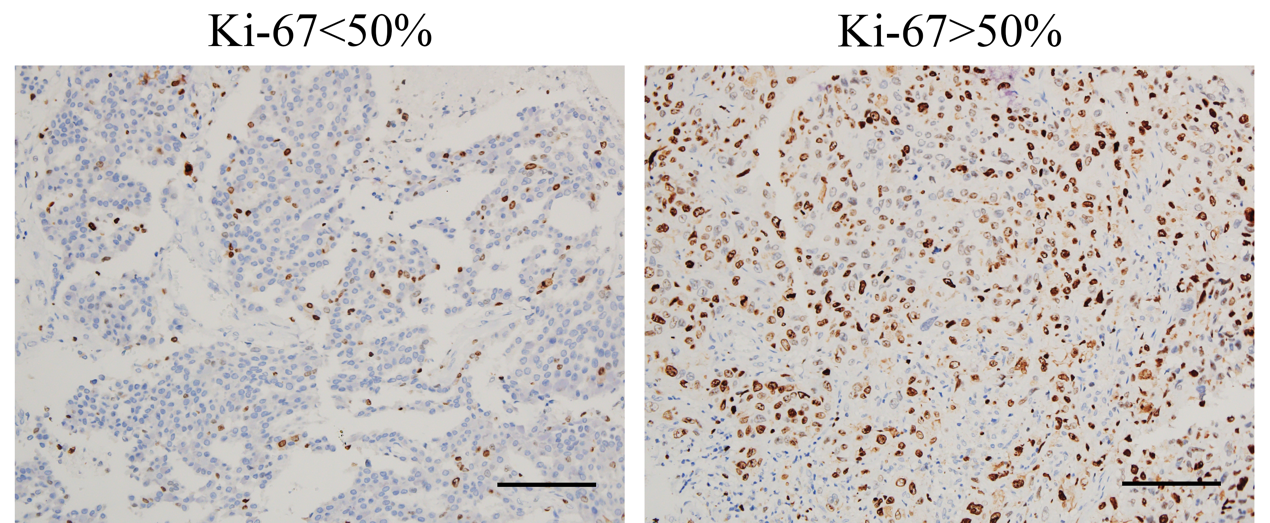


Supplementary figure 2:

Representative immunohistochemical staining images of all markers in brain metastases. A to M list the typical weak positive expression, moderate positive expression, strong positive expression immunohistochemical images of CTLA-4, PD-1, PD-L1, B7-H3, B7-H4, IDO1, EphA2, CD3, CD4, CD8, CD20, CD68 and CD163, respectively. Original magnification 200×. Scale bars, 100 µm.


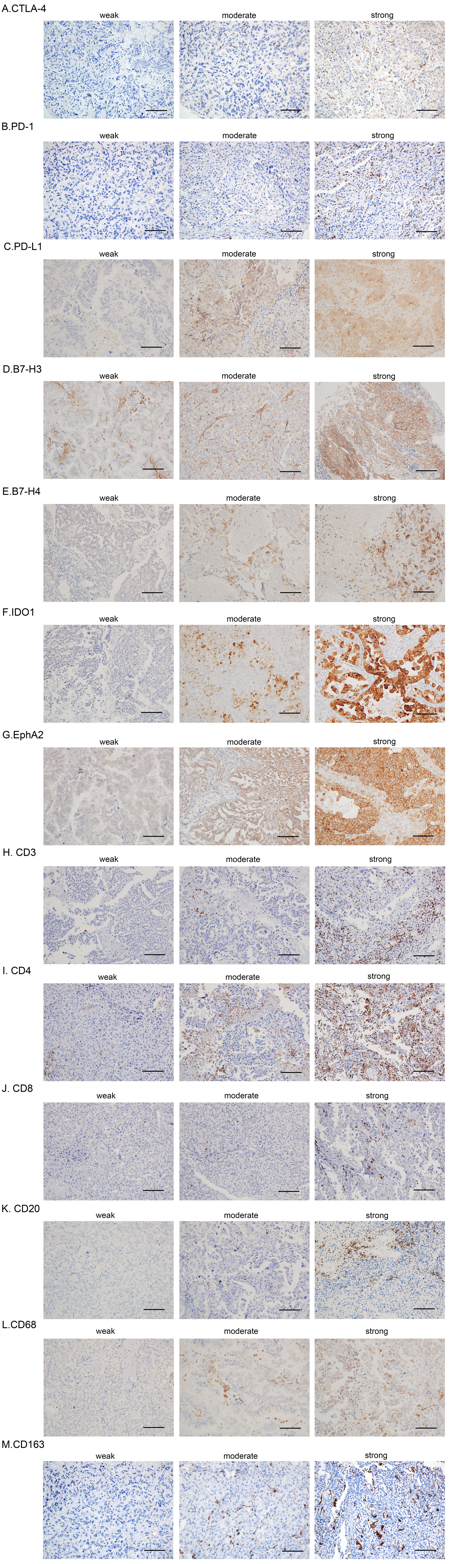


Supplementary figure 3:

Kaplan-Meier survival curves of CD3, CD4, CD8, CD20, CD163 and CD163/CD68 ratio in lung adenocarcinoma brain metastases patients. (A-F) the expression of CD3, CD4, CD8 CD20, CD163 and CD163/CD68 in stroma were not associated with patient survival (P=0.152, P=0.325, P=0.810, P=0.472, P=0.257 and P=0.202 respectively).


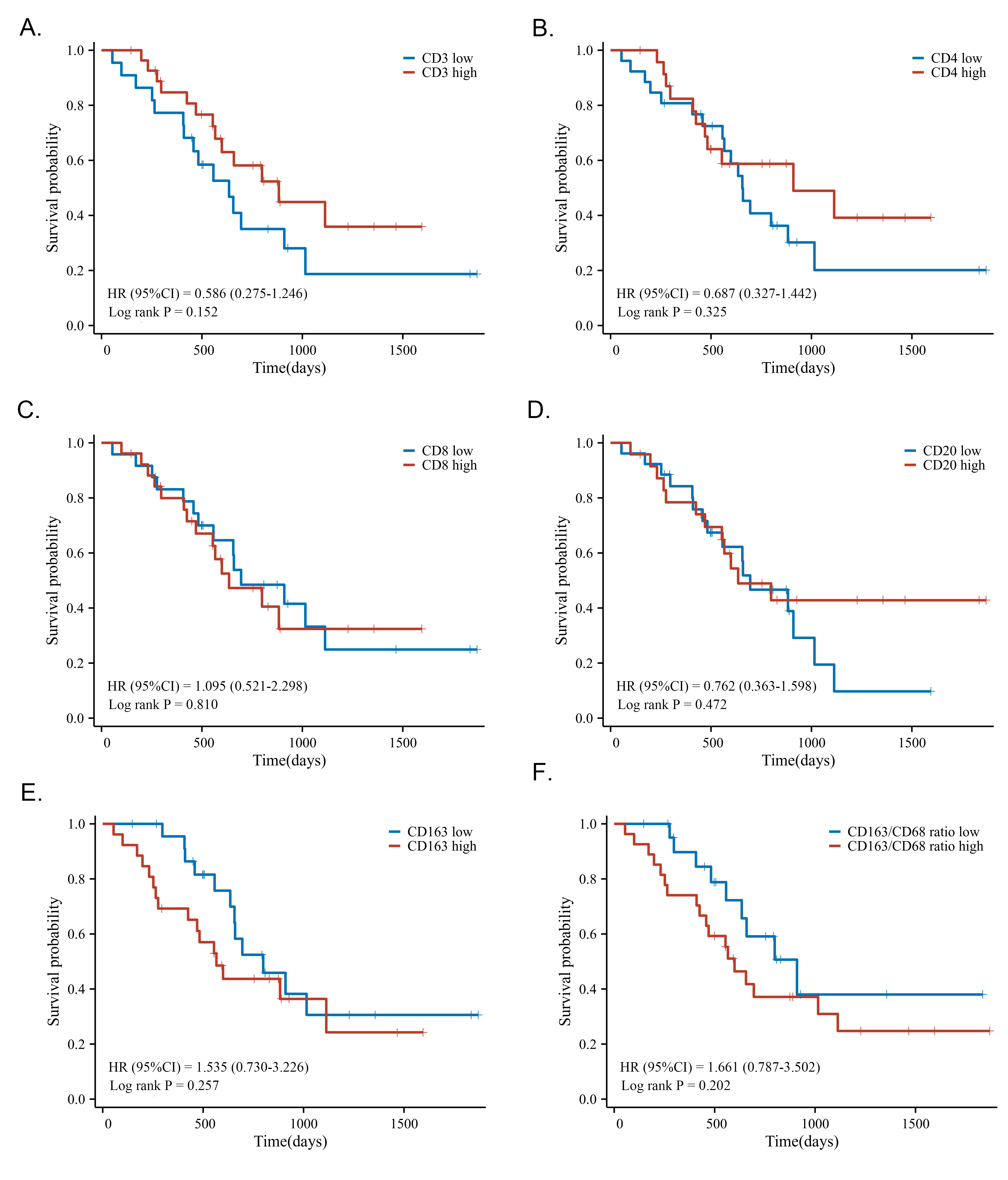


Supplementary figure 4: Kaplan-Meier survival curves of treatment modality in lung adenocarcinoma brain metastases patients (n=31). SR: surgical resection, Rad: radiation therapy, Chemo: chemotherapy, TKIs: tyrosine kinase inhibitors.


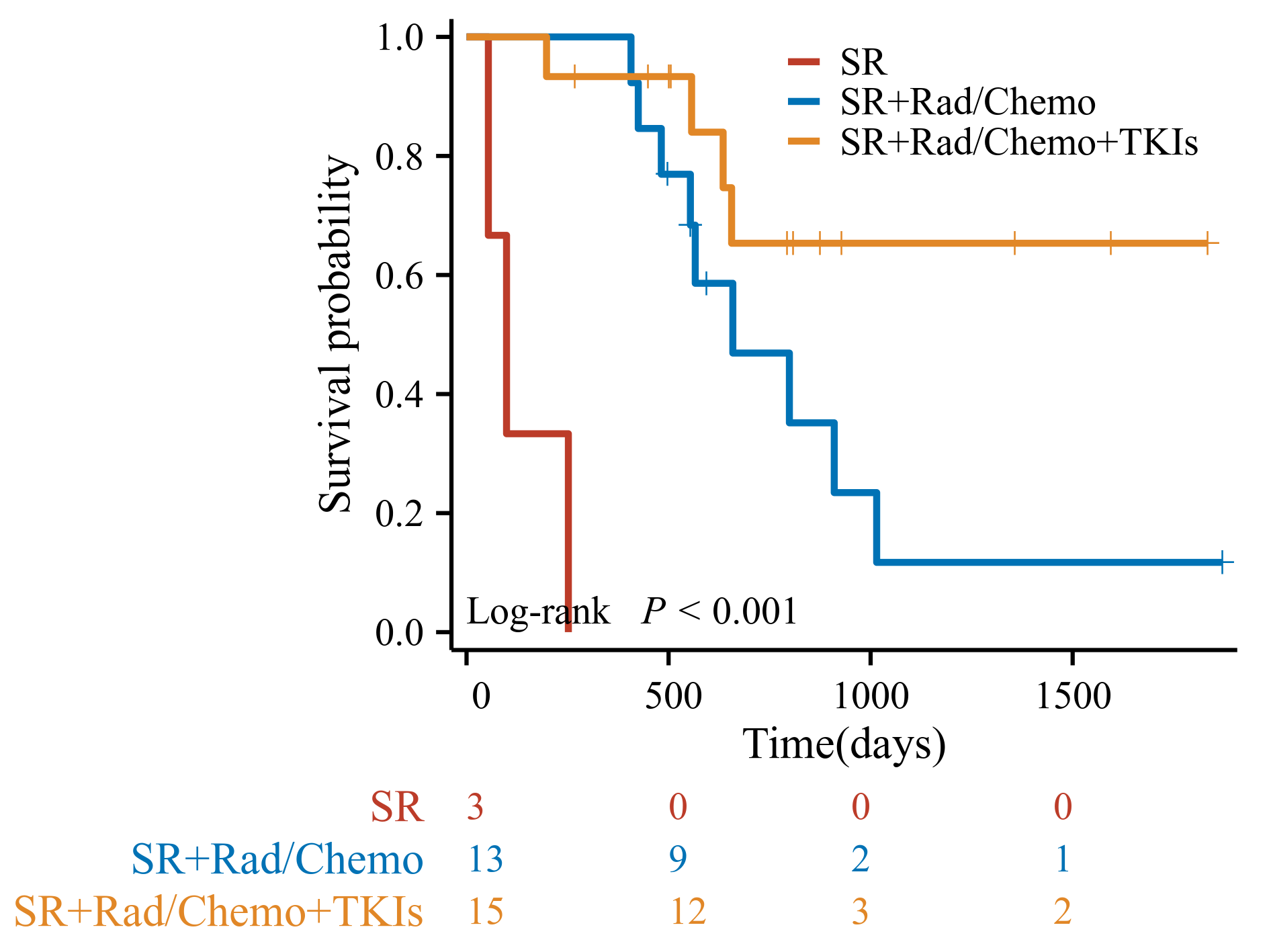

Supplement: Supplementary file 1 — Supplementary Material 1: Supplementary table 1. The immunohistochemical scores and medians of immune checkpoints, tumor-infiltrating lymphocytes, tumor-associated microglia/macrophages and tumor proliferation index Ki-67. Supplementary table 2. Infiltration of TILs in paired samples when classified by synchronous and metachronous metastasis. Supplementary table 3: Relationship between the expression of immune checkpoints and clinicopathological features in BM lesions. Supplementary table 4: Univariate analysis of prognostic factor for lung adenocarcinoma brain metastases patients. Supplementary Fig. 1: Typical immunohistochemical images of Ki-67. Original magnification 200×. Scale bars, 100 µm. Supplementary Fig. 2: Representative immunohistochemical staining images of all markers in brain metastases. A to M list the typical weak positive expression, moderate positive expression, strong positive expression immunohistochemical images of CTLA-4, PD-1, PD-L1, B7-H3, B7-H4, IDO1, EphA2, CD3, CD4, CD8, CD20, CD68 and CD163, respectively. Original magnification 200×. Scale bars, 100 µm. Supplementary Fig. 3: Kaplan-Meier survival curves of CD3, CD4, CD8, CD20, CD163 and CD163/CD68 ratio in lung adenocarcinoma brain metastases patients. (A-F) the expression of CD3, CD4, CD8 CD20, CD163 and CD163/CD68 in stroma were not associated with patient survival (P = 0.152, P = 0.325, P = 0.810, P = 0.472, P = 0.257 and P = 0.202 respectively). Supplementary Fig. 4: Kaplan-Meier survival curves of treatment modality in lung adenocarcinoma brain metastases patients (n = 31). SR: surgical resection, Rad: radiation therapy, Chemo: chemotherapy, TKIs: tyrosine kinase inhibitors [file 12885_2024_11875_MOESM1_ESM.docx]
